# Induced Pluripotent Stem Cells Show Metabolomic Differences to Embryonic Stem Cells in Polyunsaturated Phosphatidylcholines and Primary Metabolism

**DOI:** 10.1371/journal.pone.0046770

**Published:** 2012-10-15

**Authors:** John K. Meissen, Benjamin T. K. Yuen, Tobias Kind, John W. Riggs, Dinesh K. Barupal, Paul S. Knoepfler, Oliver Fiehn

**Affiliations:** 1 University of California Davis Genome Center, University of California Davis, Davis, California, United States of America; 2 Department of Cell Biology and Human Anatomy, University of California Davis School of Medicine, Davis, California, United States of America; Mayo Clinic, United States of America

## Abstract

Induced pluripotent stem cells are different from embryonic stem cells as shown by epigenetic and genomics analyses. Depending on cell types and culture conditions, such genetic alterations can lead to different metabolic phenotypes which may impact replication rates, membrane properties and cell differentiation. We here applied a comprehensive metabolomics strategy incorporating nanoelectrospray ion trap mass spectrometry (MS), gas chromatography-time of flight MS, and hydrophilic interaction- and reversed phase-liquid chromatography-quadrupole time-of-flight MS to examine the metabolome of induced pluripotent stem cells (iPSCs) compared to parental fibroblasts as well as to reference embryonic stem cells (ESCs). With over 250 identified metabolites and a range of structurally unknown compounds, quantitative and statistical metabolome data were mapped onto a metabolite networks describing the metabolic state of iPSCs relative to other cell types. Overall iPSCs exhibited a striking shift metabolically away from parental fibroblasts and toward ESCs, suggestive of near complete metabolic reprogramming. Differences between pluripotent cell types were not observed in carbohydrate or hydroxyl acid metabolism, pentose phosphate pathway metabolites, or free fatty acids. However, significant differences between iPSCs and ESCs were evident in phosphatidylcholine and phosphatidylethanolamine lipid structures, essential and non-essential amino acids, and metabolites involved in polyamine biosynthesis. Together our findings demonstrate that during cellular reprogramming, the metabolome of fibroblasts is also reprogrammed to take on an ESC-like profile, but there are select unique differences apparent in iPSCs. The identified metabolomics signatures of iPSCs and ESCs may have important implications for functional regulation of maintenance and induction of pluripotency.

## Introduction

Embryonic stem cells (ESCs) possess substantial potential for use in regenerative medicine therapies due to their capacity for unlimited self-renewal and their pluripotency to differentiate into all cell types except extraembryonic tissues. However, several challenges face the potential use of ESCs for therapies, including their potential immune rejection as allotransplants. Subsequently, the discovery that introduction of the Oct3/4, Sox2, c-Myc, and Klf4 transcription factor encoding genes could reprogram somatic cells to exhibit the morphology, growth properties, and gene expression of ESCs greatly expanded the possibilities of stem cell-based treatments and potentially resolved some of the key issues with ESC [1], [2], [3], [4], [5], [6]. Since their discovery in 2006, these cells, termed induced pluripotent stem cells (iPSCs), have been studied extensively, particularly to examine the degree to which they resemble ESC [7], [8], [9].

The high degree of similarity between ESCs and iPSCs has been confirmed on many levels [8], [9]. For example, tetraploid complementation experiments with iPSCs generating full-term mice demonstrated similarity in developmental potential and pluripotency [10], [11], [12]. Gene expression analysis, including broad profiling and focused analysis of characteristic ESC genes, as well as epigenetic studies further supported a strikingly high degree of similarity between iPSCs and ESCs [1], [2], [3], [4], [5], [6]. However, several of these reports identified a small range of differences between the two pluripotent stem cell types, illustrating that while these cells are similar, they are not identical. Some published reports suggest iPSCs are their own unique subtype of pluripotent cell with a distinguishable gene expression profile [13], and that iPSCs retain epigenetic properties or “memory” from their parental cells of origin [14]. A recent publication reported immune rejection of teratomas formed from iPSCs in contrast to teratomas formed from mouse ESCs (mESCs) [15]. In addition, in some cases iPSCs may possess a few mutations of currently unknown function [16], [17], [18], [19].

One important, but relatively less explored area related to pluripotency and stem cell biology is cellular metabolism. Metabolomics has significant potential to advance our understanding of stem cells and their relationship to other cell types, as it effectively characterizes the biochemical state of a particular cell type and delineates relevant changes compared to other experimental conditions [20]. The metabolome consists of an immense number of components connected by many complex biological pathways spanning a wide array of structural classes such as lipids, carbohydrates, amino acids, and nucleotide structures. This chemical diversity presents a particular challenge as a single analytical method cannot encompass metabolites with such diverse chemical properties [21], [22]. Differences in metabolic oxidation states were reported between mESCs and differentiated cells [23], and changes in amino acid and carbohydrate metabolism were found in mesenchymal stem cells relative to glioma cells [24]. Recently, the metabolic relationships between iPSCs, parental fibroblasts, and ESCs demonstrated that iPSC reprogramming resulted in metabolite profiles with a high degree of similarity to mESCs [25]. Specific metabolites including free fatty acids and S-adenosylmethionine cycle members displayed statistically different levels between pluripotent cell lines; hydroxyl acid levels were statistically different when comparing pluripotent cell lines to fibroblasts. Here, we employed a different metabolomics approach to evaluate and compare the metabolite profiles of iPSCs relative to parental mouse embryonic fibroblast cells (m15) and mESCs using a previously characterized iPSC line [26]. Specifically, we applied four different metabolomics platforms for analysis: direct infusion nanoelectrospray ionization mass spectrometry (nanoESI-MS), gas chromatography time-of-flight mass spectrometry (GC-TOF MS), and hydrophilic interaction (HILIC) and reverse phase (RP) chromatography quadrupole time-of-flight mass spectrometry (HILIC/RP-QTOF MS), to cover a greater extent of the stem cell metabolome. When integrating the data from these platforms based on chemical and biological similarity, resultant metabolic interaction network graphs support the notion that iPSCs have undergone near complete metabolome reprogramming, with some important metabolic changes between iPSCs and mESCs which differed from those previously reported [25].

## Results

### Direct infusion shotgun lipidomics illustrates similarity of mESC and iPSC lipid phenotypes

To begin characterizing the metabolome of mESCs and iPSCs, we employed nanoelectrospray ion trap tandem mass spectrometry (nanoESI-MS) for a first-screen metabolic investigation of the cell extracts, focusing on a rapid comparison of lipid profiles. Overall, nanoESI-MS yielded approximately 600 distinguishable ions per mass spectrum when processed with the GeneData Expressionist Refiner MS tool; individual ions were selected for structural investigation by collision induced dissociations. In direct infusion mass spectrometry, lipids are not separated by chromatography causing some level of ambiguity for annotating isobaric lipids and some lipid adduct species. Nevertheless, 157 different lipid structures including phosphatidylcholines (PC), phosphatidylethanolamines (PE), triacylglycerols (TG), diacylglycerols (DG), phosphatidylserines (PS), sphingomyelins (SM), and acylcarnitines with a diverse assortment of acyl structures were annotated by matching experimental to predicted mass spectral fragmentations by the in-house LipidBlast database, based on their major structural features such as lipid head groups and acyl chains (Table S1).

**Figure 1:**
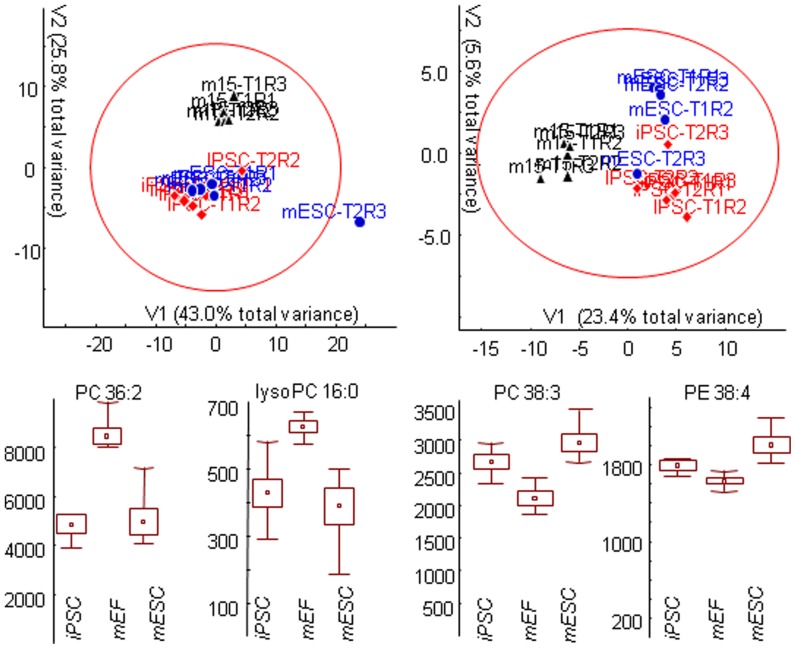
Lipidomic analysis of pluripotent cell lines (iPSC, mESC) and mouse embryonic fibroblasts (mEF) using nanoelectrospray-linear ion trap MS/MS. Upper panel: unsupervised Principal Component analysis (left) and supervised Partial Least Square regression analysis (right). Multivariate vectors with percent total variance explained. Lower panel: examples of differentially regulated membrane lipids. PC = phosphatidylcholines, PE = phosphatidylethanolamine, with number of carbons followed by number of double bonds. Mean ion intensities ± standard errors (boxes) and non-outlier ranges (whiskers).

Ion abundances were normalized to total intensity for each sample and used to determine which cell types were closest to each other in overall lipid ratios and diversity. While measurements of individual lipids in nanoESI-MS might be susceptible to ionization suppression or be obscured by isobaric interferences, use of direct infusion full scan mass spectral fingerprints is acceptable for defining the degree of between-group metabolic differences and has been shown as a suitable tool in lipid research [27], specifically because phospholipids are ideal molecules for electrospray ionization. Hence, we have used these lipidomic fingerprints as input for multivariate statistics to determine differences in lipid phenotypes between stem cells and m15 fibroblasts. Indeed, unsupervised Principal Component Analysis (PCA) of lipidomic fingerprints clearly separated m15 fibroblast cells from the lipid signatures of pluripotent cells (Figure 1). This finding supports the notion that major metabolic reprogramming events take place on the level of membrane lipids. In fact, PCA analysis of primary metabolism data confirmed that metabolic reprogramming in pluripotent stem cells extends beyond membrane lipids (Figure S1). As predicted, iPSC lipidomic and metabolomic phenotypes were so similar to mESCs that supervised techniques had to be used to distinguish the iPSC from mESC clusters. Partial least square multivariate analysis (PLS) showed that there are discernible metabolic differences between iPSCs and mESCs for both lipids and primary metabolites in addition to the dominant metabolic reprogramming from fibroblast to pluripotent cells (Figure 1 and Figure S1). iPSCs are known to possess many of the properties of ESCs, yet still possess distinguishing characteristics [8], [9] which are also reflected at the metabolic level in our results as the two cell types expressed characteristic differences in overall lipid phenotypes. This finding supports the underlying hypothesis that the overall similarity in biological behavior and gene expression of mESCs and iPSCs is also reflected in overall metabolic similarities, specifically in the diversity of lipid structures that define membranes and that are known to exert potent signaling function in cell biology [28]. While lipid fingerprinting enabled a rapid and meaningful comparison of overall metabolic characteristics, the shortcomings of direct infusion mass spectrometry necessitated the application of more detailed quantitative comparisons by superior, yet more time consuming, chromatography-based MS techniques.

### Stem cell metabolomics requires more than one platform for comprehensive annotation

**Figure 2:**
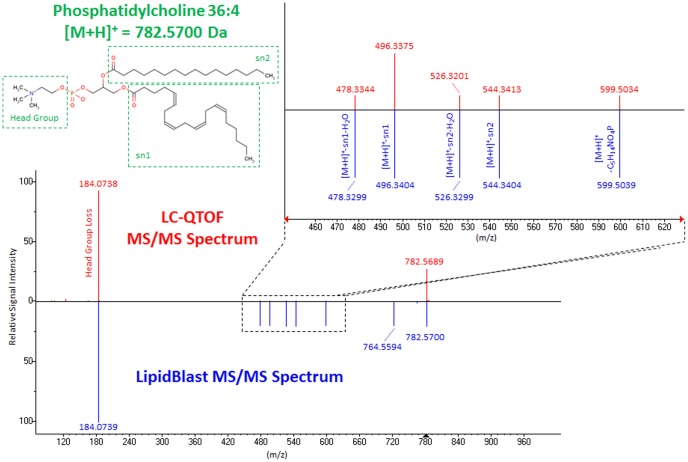
Annotation of complex lipids by tandem mass spectrometry (MS/MS) and the LipidBlast mass spectral library. LipidBlast MS/MS spectra were modeled from fragmentation spectra of authentic reference standards using computational scaffolds that altered acyl chain lengths and degree of unsaturation for each lipid class. Shown here as example is the annotation of the experimental MS/MS spectrum of arachidonyl-palmitoyl phosphatidylcholine (PC 36:4) by matching major precursor and fragment ions as well as low abundant fragments between m/z 478–599 using LipidBlast.

In order to detail the differences in lipids between stem cell types observed by nanoESI-MS fingerprinting, we profiled all three cell lines by hydrophilic interaction chromatography (HILIC) -accurate mass quadrupole time-of-flight (QTOF) tandem mass spectrometry (MS/MS). This technique yields more accurate lipid annotations because each annotated compound must fit the predicted accurate mass with a less than 1.5 mDa error, in addition to chromatographic separation of isobaric interferences and to matching the modeled LipidBlast mass spectral fragmentations (Figure 2). Furthermore, QTOF mass spectrometry is not limited by the 1/3 mass exclusion rule, unlike ion trap MS/MS, enabling the direct detection of low m/z fragment masses such as choline head groups. However, the nanoESI-ion trap MS/MS enables generation of more comprehensive fragmentations on many more precursor ions during infusions, and hence, yields more annotated lipid structures than single injection HILIC-QTOF MS/MS. Direct comparison of lipids that were analyzed by LC-QTOF MS or by nanoESI-ion trap MS/MS methods resulted in high concordance in quantitative levels of lipids between all three cell types, except for a few lipids that occurred in different isobaric or isomeric forms and which hence need LC-separation prior to detection (Figure S2).

HILIC-QTOF methodology provided data enabling annotation of many structures beyond lipids, and combined HILIC and reversed-phase liquid chromatography QTOF data sets, processed with METLIN, NIST, and LipidBlast MS/MS libraries [29], yielded a total of 55 annotated structures (Table S2). Liquid chromatography, even when used with particle sizes of less than 2 µm, still provides more selectivity (and less universality) and lower total peak capacity than classic gas chromatography (GC), especially for sugars and sugar derivatives. After derivatization and GC-TOF mass spectrometry data acquisition, the in-house BinBase data processing system [30] yielded 111 different identified and quantified structures including glycolysis, pentose phosphate pathway, and citric acid cycle metabolites as well as amino acids, free fatty acids, and sugar alcohols. However, complex and thermolabile compounds like membrane lipids or cations like acylcarnitines are not amenable to gas chromatography. Therefore, combining data from LC-QTOF MS methods with different chromatographic separation techniques and GC-TOF MS technology expanded the structural coverage of the stem cell metabolomes beyond the capacity of a single system. As expected, metabolites that were detected by both LC-QTOF MS and GC-TOF MS based methods provided similar quantitative results (Table S2), proving that data can be integrated into comprehensive metabolomic maps without quantitative distortions caused by one of our validated metabolomic platforms.

### Degree of fatty acyl unsaturation in complex lipids is a defining characteristic for stem cells

**Figure 3:**
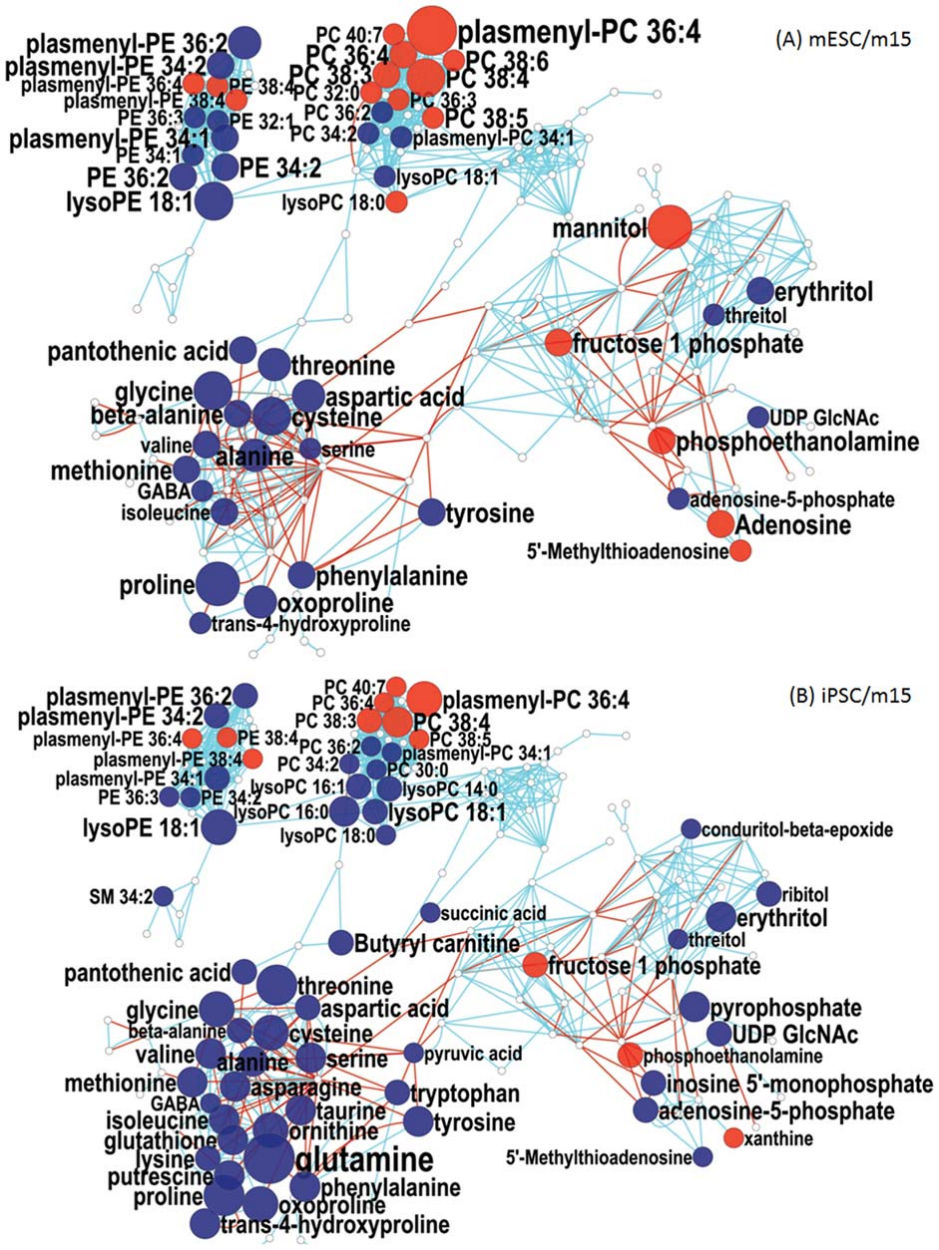
MetaMapp visualization of metabolic changes in stem cells relative to m15 fibroblast cells. Red nodes represent metabolites with increased signal intensity in stem cells; blue nodes represent metabolites with decreased signal intensity in stem cells (p<0.05). White nodes represent detected metabolites without statistically significant changes. Node sizes scale with fold change. Blue edges connect metabolites with Tanimoto chemical similarity >700; red edges connect reaction-pair metabolites from the KEGG RPAIR database. (**A**): MetaMapp network comparing mESCs to m15 fibroblasts. (**B**): MetaMapp network comparing mESCs to m15 fibroblasts.

**Figure 4:**
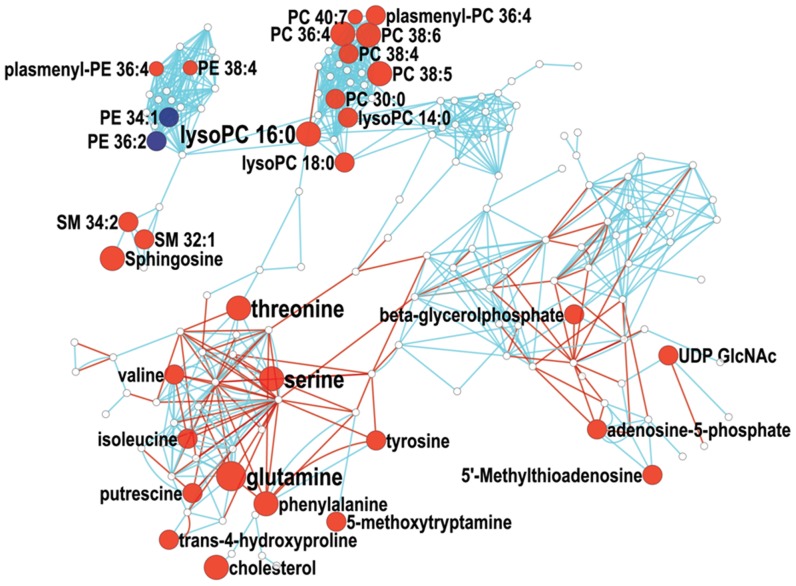
MetaMapp visualization of metabolic changes in mouse embryonic stem cells relative to metabolite levels in induced pluripotent stem cells. Red nodes represent metabolites with increased signal intensity in stem cells; blue nodes represent metabolites with decreased signal intensity in stem cells (p<0.05). White nodes represent detected metabolites without statistically significant changes. Node sizes scale with fold change. Blue edges connect metabolites with Tanimoto chemical similarity >700; red edges connect reaction-pair metabolites from the KEGG RPAIR database.

In order to reveal differences in metabolic regulation between the cell types used in this study, we mapped all identified compounds from LC-QTOF and GC-TOF MS data onto metabolite network graphs that were generated by the MetaMapp tool which integrates both biochemical and chemical similarity (Figures 3,4) [31], [32]. Interestingly, the degree of desaturation of phosphatidylcholine (PC) membrane lipids was different when comparing mESCs to m15 fibroblast cells (Figure 3A). All seven detected PC structures with three or more double bonds were detected at statistically significant elevated levels in mESCs, and all PCs with one or two double bonds were less abundant in mESC though only two of six presented a p<0.05. This result correlates with previously reported observations of higher levels of unsaturated structures, including PCs, in mESCs [23]. Complementing this report, our results now provide evidence that these elevated levels of highly unsaturated structures only affect phosphatidylcholines, as phosphatidylethanolamines (PE), the second largest class of complex membrane lipids revealed in our annotations, did not display the same compositional difference observed for PCs. Only one highly unsaturated PE and two plasmenyl-PEs with four or more double bonds were observed at statistically significant higher levels, out of a total of nine highly unsaturated PE and plasmenyl-PE structures. In contrast, seven out of eight more saturated PE and plasmenyl-PE lipids with one to three double bonds were less abundant in mESCs compared to the membrane lipid compositions in fibroblasts (*p*<0.05). Phosphoethanolamine, a biosynthetic precursor for PEs [28], was also elevated in mESCs. A direct comparison of complex lipids with the same acyl chains confirmed this differential regulation of classes of membrane lipids: PC 36:3 (18:2/18:1) was 53% higher in mESCs compared to fibroblasts, while PE 36:3 (18:2/18:1) was found to be 33% lower in mESCs. The detected sphingomyelins with one or two double bonds were not found differentially expressed in these comparisons.

The effect of metabolic reprogramming of membrane lipids in mESCs was also seen in in the comparison of iPSCs to m15 fibroblasts (Figure 3B). Highly unsaturated PC and plasmenyl-PC structures with three or more double bonds increased in iPSCs, while less unsaturated PCs and plasmenyl-PCs with one or two double bonds were found at decreased levels. The characteristic differential reprogramming of PEs and PCs in mESCs was also confirmed for iPSCs. Highly unsaturated PE structures did not display the same broad increase observed in PCs, and PE and plasmenyl-PE structures with one to three double bonds decreased. Phosphoethanolamine levels increased in iPSCs while SMs were found largely unchanged.

### Down-regulation of amino acid metabolism is characteristic for pluripotent stem cells

We found that one of the most apparent features of metabolic reprogramming is the large decrease in amino acid pools in iPSCs relative to parental fibroblasts such that iPSCs are far more similar in this regard to mESC (Figure 3A,B). Changes in amino acids were not restricted to a particular subclass but spanned many different biosynthetic pathways and transport mechanisms. In comparison, changes in carbohydrate and hydroxyl acid metabolism, including citric acid cycle metabolites, were far less pronounced. Annotated structures included four glycolysis metabolites, two pentose phosphate pathway metabolites, and four citric acid cycle metabolites. None of these structures presented significant differences between mESC and m15 fibroblast cell types, and only succinic acid showed a slight decrease in concentration in iPSCs compared to m15 fibroblasts. Despite the large changes in expression of complex lipids, no change was observed in the status of free fatty acids in the compared cell types.

### iPSCs and mESCs differ in lipid profiles, amino acids, and polyamine biosynthesis metabolites

The iPSCs displayed several differences from the parental m15 fibroblast cells that were not detected in the mESC/m15 comparison (Figure 3A,B). All six lyso-PC and lyso-PE structures, representing either saturated acyl chains or acyl chains with a single double bond, were significantly decreased in iPSCs. In iPSCs, but not in mESCs, further metabolic differences with fibroblasts were apparent in polyamine biosynthesis by a down regulation of both putrescine and ornithine, a putrescine precursor, as well as 5′-methylthioadenosine, a product of polyamine biosynthesis which inhibits spermine biosynthesis downstream of putrescine in the polyamine biosynthesis pathway [33], [34]. Thirdly, purine metabolism was found to be significantly changed in iPSCs with a decrease of the nucleotide adenosine-5-phosphate and its deamination product inosine-5-phosphate which led to an increase in xanthine, a metabolite in the purine salvage pathway [35]. Together with the more pronounced decreases in amino acid metabolism, metabolic reprogramming in iPSCs had other off-target effects on pathways involved in nitrogen metabolism, namely polyamine and purine biosynthetic pathways.

We compared the iPSCs and mESCs directly to each other to obtain a better overview of changes in metabolite profiles (Figure 4). This comparison revealed substantially fewer differences than comparisons of either pluripotent stem cell line to the m15 fibroblast cell line, confirming the clustering of lipid phenotypes in the direct infusion partial least square analysis (Figure 1). Despite their overall similarity, there were some clear differences between the two pluripotent stem cell lines. While both iPSCs and mESCs displayed similar lipid profiles, the magnitude of those differences varied significantly by cell type. All eight PC and plasmenyl-PC structures with three or more double bonds were elevated in mESCs relative to iPSCs, while shorter chain, more saturated PC and plasmenyl-PC structures were present at similar levels. All PEs with one or two double bonds were less abundant in the mESCs, with more than half displaying statistical significance, while more unsaturated PEs did not display substantial differences. All reported lyso-PC structures were present at higher levels in mESCs versus iPSCs, but this effect was most pronounced and only statistically relevant in saturated lyso-PCs. Again, the appearance of 5-methylthioadenosine and putrescine implicated an effect on the polyamine biosynthesis pathway. Numerous amino acids displayed differences between the pluripotent cell types. Interestingly, the amino acids that displayed the greatest differences were those that were uncharged yet polar in nature.

These data indicate that some of the major differences in lipid profiles between pluripotent stem cells and the fibroblast cells are more pronounced in mESCs compared to iPSCs. In addition, elevated levels of 5′-methylthioadenosine and lower levels of amino acids and putrescine in iPSCs relative to mESCs suggest that while the iPSC metabolome is shifted during cellular reprogramming to be remarkably similar to that of mESCs, reprogramming primary metabolism may not be fully complete in iPSCs.

## Discussion

We here show that metabolomics can discern the effects of genetic differences of cell lines by highlighting metabolic similarities and differences of pluripotent cell types compared to somatic cells. We employed multiple mass spectrometry-based techniques to overcome analytical challenges, such as efficient throughput and metabolite coverage as all analytical methods possess coverage limitations [21], [22]. Direct infusion nanoESI-mass spectrometry enabled a rapid analysis of different lipid structures and confirmed that the pluripotent cells are strikingly similar in terms of lipid metabolite signatures relative to fibroblasts indicating near complete metabolic reprogramming of iPSCs. However, it also revealed some potentially important differences between iPSCs and the parental fibroblasts and more subtle distinctions between mESCs and iPSCs.

While the nanoESI-MS method is an effective preliminary approach to observe variation between sample groups due to speed and MS/MS coverage, it retains some limitations. The lack of chromatographic separation and low mass spectrometric resolution causes isobaric and isomeric structures to form single peaks in the mass spectra, complicating correlation of ion intensities to a single structure. Direct infusion analysis is also particularly susceptible to ion suppression, potentially compromising appropriate reflection of compound concentration [22], [36]. Application of (lower throughput) GC-TOF MS and LC-QTOF MS chromatography-based analytical methods minimized the potential impact of these limitations and expanded metabolite coverage beyond the capabilities of either individual system. The GC-TOF MS method provided the greatest number of identified compounds, which is a reflection of a highly developed infrastructure designed specifically for the applied system and method [30]. While MS/MS libraries are available for use with the LC-QTOF MS system, identification was greatly hampered by both library coverage and acquisition of MS/MS spectra. The LC-QTOF MS uses a quadrupole to isolate a particular ion for generation of MS/MS data, and if a particular ion does not achieve the relative intensity necessary to trigger isolation in a data-dependent MS/MS acquisition mode, the MS/MS data necessary for annotation will not be available. Subsequently, while the current analysis covers many structures, there are still many other metabolites that were not annotated and may provide additional insight into the metabolic state of iPSC, mESC, and m15 fibroblast cell types.

Several reports manifest large metabolic differences between fibroblast or other somatic cells and pluripotent cells, for example with respect to energy metabolism [25], [37], [38]. Metabolomic research groups have stated a range of other significant differences after nuclear reprogramming, most notably on the level of nucleotides and purines [25] and membrane lipids [23], but neither group identified metabolic differences in lactate or glucose metabolism. In accordance with these reports, we have observed very large differences in metabolic phenotypes between mouse embryonic fibroblasts and embryonic stem cells (Figure 1 and Figure S1) which mostly conferred metabolic reprogramming in amino acid and lipid metabolism, but not in compounds such as taurine, reported as being different in extracellular footprints 5–7 days after nuclear reprogramming by Folmes et al. [37]. We detected intracellular taurine levels with two independent analytical techniques, LC-QTOF MS and GC-TOF MS. Neither of these methods found quantitative metabolic differences for taurine or energy-related metabolism. Authors have noted the similarity between energy metabolism in stem cells and cancer cells with respect to the use of anaerobic glycolysis for generating ATP rather than by mitochondrial oxidation [38], similar to the well-known Warburg effect in cancer cells [39]. It has been pointed out to be of critical importance to control for available oxygen levels in cell cultures if hypotheses about hypoxic metabolism are to be tested, for example in cancer cells [40]. To our knowledge, however, none of the reports in stem cell metabolism actually monitored and controlled the level of oxygen available to cells, including results reported here.

This work detailed many metabolic similarities and differences between iPSCs, mESCs, and embryonic fibroblasts beyond those metabolites covered recently [25] by encompassing additional structures including lipids, polyamines and amino acids. While parts of the metabolome coverage were overlapping, quantitative results revealed conflicting trends to this previous publication [25]. For example, in the study presented here we did not find any increase in free fatty acids, unlike a seven-fold difference reported before between different pluripotent cell lines [25], and we also could not confirm broad changes in hydroxyl acid levels when comparing pluripotent cell lines to fibroblasts. While we did observe statistically significant changes in 5′-methylthioadenosine levels in iPSCs relative to ESCs, the effect was less than two-fold and in the opposite direction from the previously published analysis [25].

It is unlikely that conflicting results between our data and the recently published study [25] are due to the analytical methodologies used in our research, because we have utilized the broadest line of technologies that were yet applied to stem cell metabolism. NanoESI-MS, GC-TOF MS, and HILIC- and RP liquid chromatography-QTOF MS based technologies facilitated expansion of metabolite profiling to encompass a chemically diverse array of cellular metabolites and enabled an in-depth investigation and comparison of iPSC, m15 fibroblast, and mESC metabolite profiles. Application of this global approach delineated some of the features which, despite their similarity, distinguish iPSCs from mESCs and identified candidate mechanisms that may be subject to more targeted, focused methods to attain a more clear understanding of potential cause and biological impact. Additional studies of metabolic phenotypes and genomic differences of iPSCs, ESCs, and embryonic fibroblasts are critical to further advances in our understanding of pluripotent cell metabolism.

Differences in the degree and range of detected metabolic changes in stem cell reprogramming may not only be founded in differences in cell culture conditions, but may also relate to the differences in cell lines investigated. For example, line-to-line variability in induced pluripotent stem cells has also been demonstrated repeatedly; for example, different ways to derive iPS-cells directly influence x-inactivation and epigenetic variability [41], [42]. In analogy to the apparently conflicting results on stem cell metabolism, proteomics analyses also showed substantial differences between each iPS cell line studied, in addition to differences between individual embryonic stem cell lines [43]. Overall, all reports so far concur that metabolic reprogramming is a key feature in reprogramming cells into a pluripotent state which may involve a wide range of metabolic pathways. The extent of which pathways are mostly reprogrammed appear to be dependent on the actual culture conditions, time points and cell types being studied. Indeed, taking together these different reports, metabolomics may be a tool to determine the phenotypic proximity of different iPSC lines to embryonic stem cells, in addition to informing about metabolic reprogramming events from fibroblast cells.

An area of intense interest in the stem cell field has been defining the properties of iPSCs relative to ESCs. Just how similar are iPSCs and ESCs? Based on previous work at the cell biological, gene expression, and epigenetic levels, the two pluripotent stem cell types are remarkably similar, although not identical. We detected higher levels of phosphatidylcholines comprised of three or more double bonds in mESCs relative to m15 fibroblasts, confirming recently reported results [23]. Such increase in unsaturation levels of polyunsaturated PCs may alter membrane fluidity and hence can contribute to physiological changes that are relevant for stem-cell phenotypes. However, the more detailed investigation presented here also reveals that this up-regulation of membrane lipids did not extent to phosphatidylethanolamines in either pluripotent cell type. Indeed, sometimes just the opposite regulation was found as for the 18:1/18:2 PC and 18:1/18:2 PE lipids, especially when comparing mESCs to fibroblasts. iPSCs displayed the same tendency, supporting the concept that the iPSC model reflects ESCs in terms of regulating membrane composition. Such differential changes may also be due to different activity levels of methylating enzymes, because PCs may originate from PEs by methylation steps catalysed by the enzyme phosphatidylethanolamine N-methyltransferase, a ∼20 kDa transmembrane-spanning enzyme located mainly in the endoplasmic reticulum. PCs may otherwise originate *de novo* from CDP-choline and diacylglycerols, but the exact origin of PCs in these cell types has not been determined yet. DNA- and Histone-methyltransferase activities are well known to be involved in stem cell developmental phenotypes [44]. It is interesting that the magnitude of PE-dependent methyltransferase activity was less pronounced in iPSCs than in mESCs, suggesting the genetic reprogramming of the iPSC line was not entirely adequate to yield identical mESC lipid profiles.

Metabolic differences between the iPSC and mESC lines were also evident in primary metabolism, specifically for amino acids. Both cell types yielded lower amounts of free amino acids compared to the m15 fibroblasts, but several amino acids were present at even lower levels in iPSCs relative to mESCs. While this dissimilarity might point to differences in protein synthesis (i.e. differences in amino acid consumption), differences in amino acid transporter activities would be another potential explanation given that both essential and non-essential polar uncharged amino acids displayed the most substantial differences. Statistically significant changes in both putrescine and 5-methylthioadenosine indicate that the polyamine biosynthesis pathway is another deviation between mESCs and iPSCs. This effect is notable due to polyamine biosynthesis involvement in cell proliferation and differentiation [45].

The body of literature in stem cell metabolism is growing. However, at this point mechanistic interpretations have not established which parts of metabolic reprogramming are a necessary condition for pluripotency and which metabolic differences are consequence rather than causal factor in cellular de-differentiation. Detailed investigations on mechanistic aspects of stem cell metabolism, including flux studies and studies under hypoxia versus normoxic conditions, may be needed to reveal the underlying metabolic prerequisites for pluripotency.

## Materials and Methods

Cell material was developed, cultured, and characterized as described previously [26]. Mouse embryonic fibroblast and pluripotent cells were maintained with identical culture media (ES cell media). The induced pluripotent stem cells showed both cellular phenotypic and gene expression profiles similar to levels observed in mouse embryonic stem cells. To prepare cell pellets for metabolite extraction, all cells were harvested by trypsinization, washed three times with cold PBS, divided into one million cell aliquots, and flash frozen in liquid N_2_. Cell pellets were stored at −80°C prior to extraction.

Three replicates from each cell type were used for nanoESI-MS analysis. Cellular metabolites were extracted with 225 µL of methanol, 750 µL of t-butyl methyl ether, and 187.5 µL of H_2_O. Mass spectrometry analysis was performed with a LTQ linear ion trap mass spectrometer (ThermoFisher Scientific, San Jose, CA) coupled to an Advion NanoMate chip based nanoelectrospray ionization source (Advion Biosciences Inc., Ithaca, NY). Sample material was analyzed in positive ion mode and MS/MS acquisition was applied to pooled samples representative of each experimental condition. Data files were processed with GeneData Expressionist Refiner MS v6.2.0 software (GeneData, Basel, Switzerland), available at http://www.genedata.com/products/expressionist.html) and processed with Statistica 9.0 software (StatSoft, Tulsa, OK) for PLS analysis. The NIST MS Search program (National Institutes of Standards and Technology, Gaithersburg, MD) was used to compare MS/MS data to LipidBlast, an in-house library of lipid structure MS/MS spectra.

Six replicates of each sample condition were extracted, derivatized, and analyzed as reported previously [30], [46] for GC-TOF analysis. Sample materials were analyzed with a Leco Pegasus IV time of flight mass spectrometer (Leco Corporation, St. Joseph, MI) coupled to an Agilent 6890 gas chromatograph (Agilent Technologies, Santa Clara, CA) equipped with a 30 m long 0.25 mm i.d. Rtx5Sil-MS column and a Gerstel MPS2 automatic liner exchange system (Gerstel GMBH & Co.KG, Mülheim an der Ruhr, Germany). Result files were exported to our servers and further processed by our metabolomics BinBase database [47].

Six replicates of each sample condition were extracted with 3∶1 methanol∶H_2_O for LC-QTOF MS analysis. An Agilent 1200 Series HPLC system equipped with either a Waters 1.7 µm Acquity BEH HILIC 2.1×150 mm column (Waters Corporation, Milford, MA) or an Agilent 1.8 µm Zorbax Eclipse Plus C18 2.1×150 mm column were used for chromatographic separations. LC eluents were analyzed with an Agilent 6530 accurate-mass Q-TOF mass spectrometer equipped with an Agilent Jet Stream ESI source in positive ion mode. MS and MS/MS data was collected and mass calibration was maintained by constant infusion of reference ions. For MS/MS library annotation, raw data files were compared to METLIN, LipidBlast, and NIST MS/MS libraries. All MS/MS library matches were manually confirmed.

Annotated structures were clustered with a web-based PubChem structural clustering tool and were used as an input in MetaMapp software (available at: http://metamapp.fiehnlab.ucdavis.edu) with CID-KEGG ID pairs for generation of Cytoscape network files [48]. Results of differential statistics generated using Statistica 9.0 software were converted into Cytoscape node attribute files and were imported into Cytoscape. The graph was visualized using an organic layout algorithm in Cytoscape.

A more detailed summary of procedures is available as Table S3.

## Supporting Information

Figure S1 **Principal Component Analysis (PCA) and Partial Least Square (PLS) multivariate analysis on all three metabolomic platforms combined (left panels) or excluding the nanoelectrospray-ion trap MS data (right panels).**
(PDF)

Figure S2 **Method comparison for quantification of select compounds in m15 progenitor cells, induced pluripotent stem cells and embryonic stem cells, detected in more than one metabolomics platform.** Upper panel: unsaturated phosphatidyl-lipids detected by HILIC-QTOF MS and by nanoelectrospray-linear ion trap mass spectrometry. All data are given as normalized intensities. For method details, see *Supplement Methods*. Lower panel: quantification of pantothenic acid by gas chromtography (GC)-time of flight mass spectrometry (TOF), hydrophilic interaction chromaography/quadrupole time pf flight mass spectrometry (QTOF) and reversed phase liquid chromatography-QTOF.
(PDF)

Table S1 **Identified lipids by shotgun lipidomics using direct infusion nanoelectrospray/linear ion trap tandem mass spectrometry and LipidBlast annotations with manual quality checks.**
(PDF)

Table S2 **Identified metabolites in m15 and stem cell cultures using gas chromatography-time of flight mass spectrometry and BinBase annotations, and liquid chromatography-quadrupole time of flight mass spectrometry and Metlin and LipidBlast annotations.** Significance levels are given as one-way ANOVA *p*-values.
(PDF)

Table S3 **Extended information on** **Materials and Methods** **: Cell Culture and Preparation; NanoESI-MS Extraction and Analysis; GC-TOF MS Extraction and Analysis; LC-QTOF MS Extraction and Analysis; MetaMapp Network Visualization.**
(PDF)
