# Supplementary figures and images for: Induced Pluripotent Stem Cells Show Metabolomic Differences to Embryonic Stem Cells in Polyunsaturated Phosphatidylcholines and Primary Metabolism
Source: PLoS One. 2012 Oct 15;7(10):e46770. doi: 10.1371/journal.pone.0046770 (PMC3471894; doi:10.1371/journal.pone.0046770)

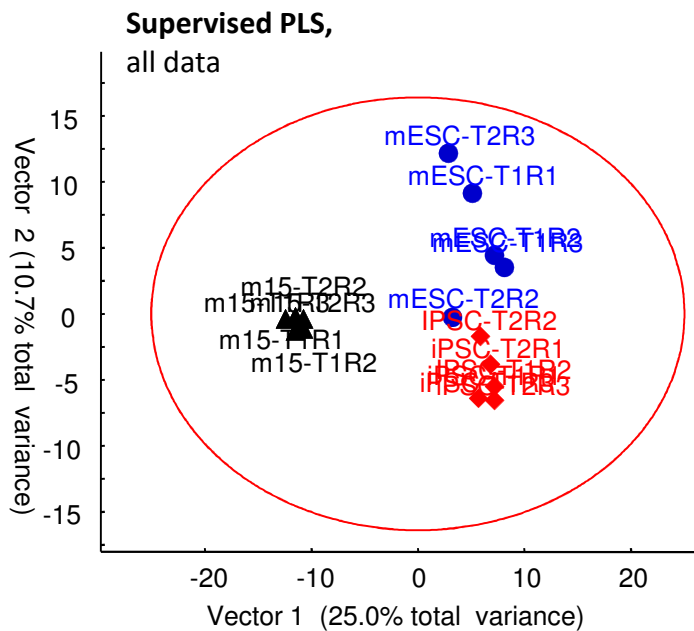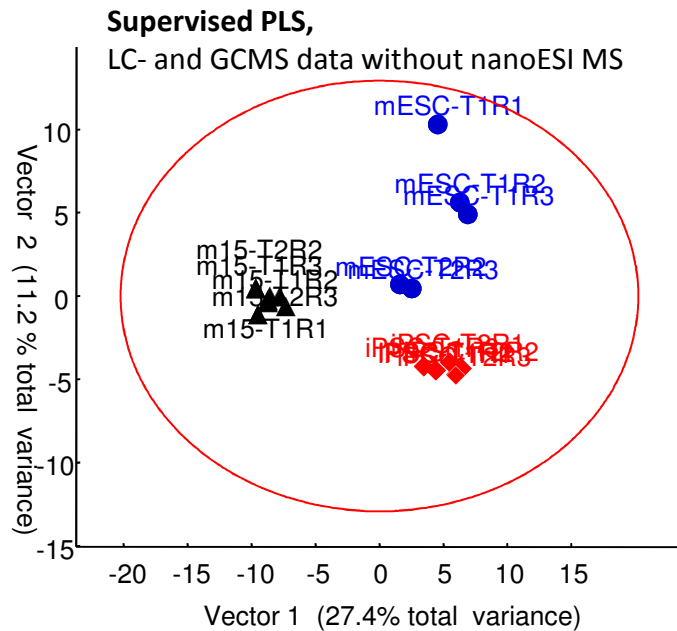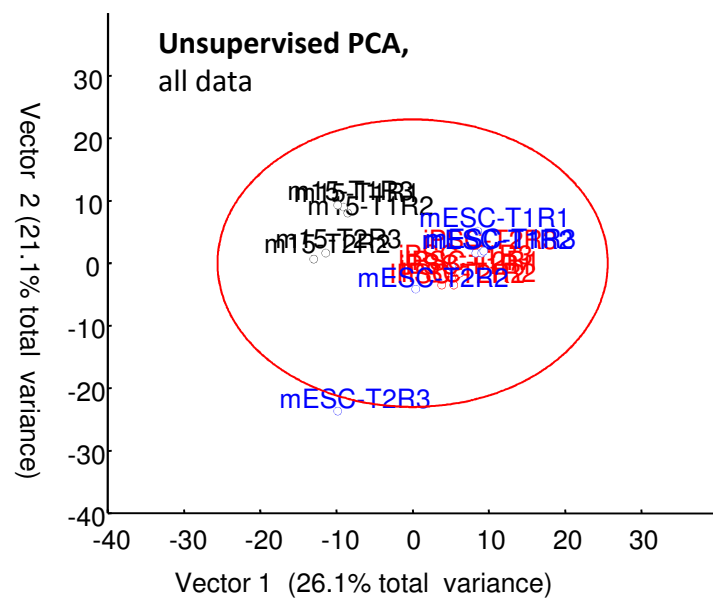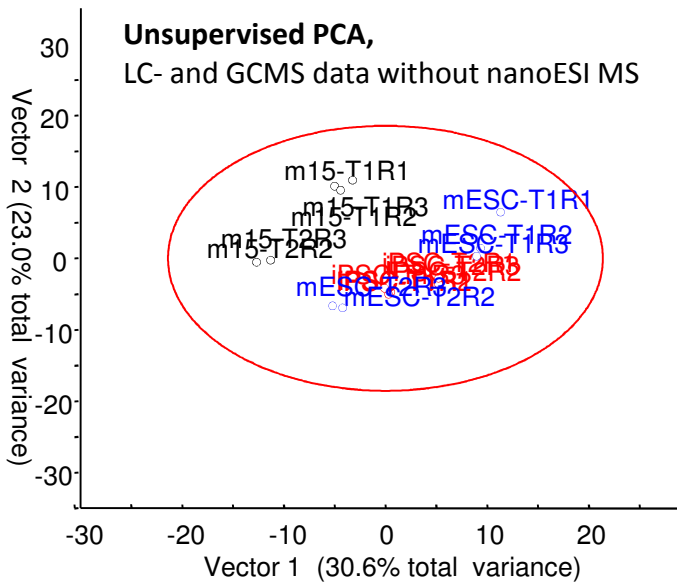

Supplement: Figure S1 — Principal Component Analysis (PCA) and Partial Least Square (PLS) multivariate analysis on all three metabolomic platforms combined (left panels) or excluding the nanoelectrospray-ion trap MS data (right panels). (PDF) [file pone.0046770.s001.pdf]

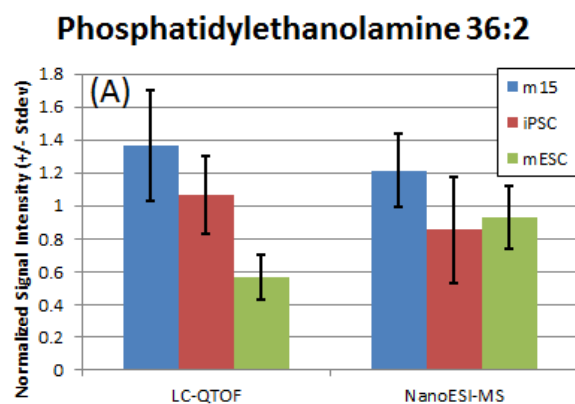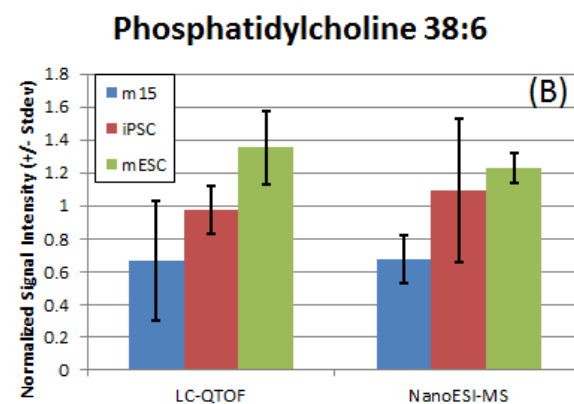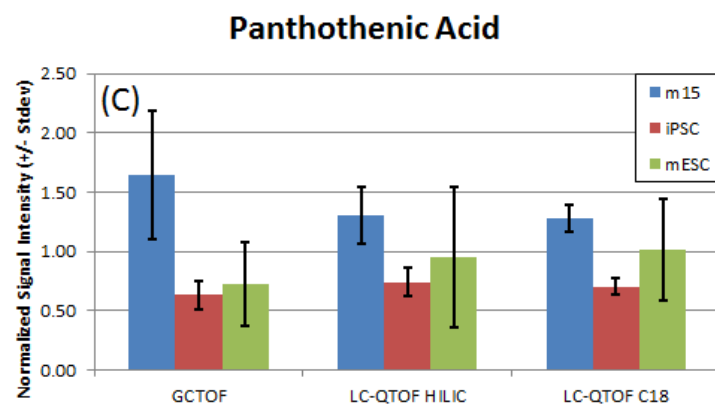

Supplement: Figure S2 — Method comparison for quantification of select compounds in m15 progenitor cells, induced pluripotent stem cells and embryonic stem cells, detected in more than one metabolomics platform. Upper panel: unsaturated phosphatidyl-lipids detected by HILIC-QTOF MS and by nanoelectrospray-linear ion trap mass spectrometry. All data are given as normalized intensities. For method details, see Supplement Methods. Lower panel: quantification of pantothenic acid by gas chromtography (GC)-time of flight mass spectrometry (TOF), hydrophilic interaction chromaography/quadrupole time pf flight mass spectrometry (QTOF) and reversed phase liquid chromatography-QTOF. (PDF) [file pone.0046770.s002.pdf]
