# Supplementary material for: Induced Pluripotent Stem Cells Show Metabolomic Differences to Embryonic Stem Cells in Polyunsaturated Phosphatidylcholines and Primary Metabolism
Source: PLoS One. 2012 Oct 15;7(10):e46770. doi: 10.1371/journal.pone.0046770 (PMC3471894; doi:10.1371/journal.pone.0046770)
Supplement: Table S1 — Identified lipids by shotgun lipidomics using direct infusion nanoelectrospray/linear ion trap tandem mass spectrometry and LipidBlast annotations with manual quality checks. (PDF) [file pone.0046770.s003.pdf]

Acylcarnitines	Phosphatidylcholines	Phosphatidylethanolamines
Acylcarnitine C12:0	Lysophosphatidylcholine C9:0	Lysophosphatidylethanolamine C16:0
Acylcarnitine C12:1	Lysophosphatidylcholine C10:0	Plasmenyl-Phosphatidylethanolamine C34:0
Acylcarnitine C16:0	Lysophosphatidylcholine C16:0	Plasmenyl-Phosphatidylethanolamine C34:1
Acylcarnitine C18:0	Lysophosphatidylcholine C18:1	Plasmenyl-Phosphatidylethanolamine C36:0
Acylcarnitine C18:1	Lysophosphatidylcholine C18:2	Plasmenyl-Phosphatidylethanolamine C36:1
Acylcarnitine C18:3	Plasmenyl-Phosphatidylcholine C30:1	Plasmenyl-Phosphatidylethanolamine C36:3
Acylcarnitine C20:2	Plasmenyl-Phosphatidylcholine C31:0	Plasmenyl-Phosphatidylethanolamine C36:4
Acylcarnitine C24:1	Plasmenyl-Phosphatidylcholine C31:1	Plasmenyl-Phosphatidylethanolamine C36:5
**Diacylglycerols**	Plasmenyl-Phosphatidylcholine C32:0	Plasmenyl-Phosphatidylethanolamine C38:0
Diacylglycerol C24:1	Plasmenyl-Phosphatidylcholine C33:0	Plasmenyl-Phosphatidylethanolamine C38:4
Diacylglycerol C39:2	Phosphatidylcholine C9:0	Plasmenyl-Phosphatidylethanolamine C38:5
Diacylglycerol C41:2	Phosphatidylcholine C15:0	Plasmenyl-Phosphatidylethanolamine C38:6
Diacylglycerol C43:4	Phosphatidylcholine C22:2	Plasmenyl-Phosphatidylethanolamine C40:0
Diacylglycerol C47:0	Phosphatidylcholine C26:0	Plasmenyl-Phosphatidylethanolamine C40:6
Diacylglycerol C48:0	Phosphatidylcholine C27:0	Phosphatidylethanolamine C12:0
Diacylglycerol C50:0	Phosphatidylcholine C28:1	Phosphatidylethanolamine C19:1
**Diacylglycerophosphates**	Phosphatidylcholine C29:2	Phosphatidylethanolamine C33:0
Diacylglycerophosphate C12:0	Phosphatidylcholine C30:0	Phosphatidylethanolamine C33:1
Diacylglycerophosphate C20:3	Phosphatidylcholine C30:1	Phosphatidylethanolamine C34:1
Diacylglycerophosphate C33:1	Phosphatidylcholine C31:0	Phosphatidylethanolamine C35:1
Diacylglycerophosphate C34:0	Phosphatidylcholine C31:3	Phosphatidylethanolamine C35:5
Diacylglycerophosphate C36:0	Phosphatidylcholine C31:5	Phosphatidylethanolamine C35:6
Diacylglycerophosphate C36:1	Phosphatidylcholine C31:6	Phosphatidylethanolamine C36:1
Diacylglycerophosphate C38:0	Phosphatidylcholine C32:0	Phosphatidylethanolamine C36:2
Diacylglycerophosphate C39:2	Phosphatidylcholine C32:1	Phosphatidylethanolamine C36:4
Diacylglycerophosphate C45:0	Phosphatidylcholine C32:2	Phosphatidylethanolamine C37:0
Diacylglycerophosphate C8:0	Phosphatidylcholine C32:5	Phosphatidylethanolamine C37:5
**Phosphatidylserines**	Phosphatidylcholine C33:0	Phosphatidylethanolamine C37:6
Phosphatidylserine C36:4	Phosphatidylcholine C33:2	Phosphatidylethanolamine C37:7
Phosphatidylserine C41:0	Phosphatidylcholine C34:0	Phosphatidylethanolamine C38:2
**Sphingomyelins**	Phosphatidylcholine C34:1	Phosphatidylethanolamine C38:4
Sphingomyelin C21:0	Phosphatidylcholine C34:2	Phosphatidylethanolamine C38:6
Sphingomyelin C31:0	Phosphatidylcholine C34:3	Phosphatidylethanolamine C38:7
Sphingomyelin C32:0	Phosphatidylcholine C35:1	Phosphatidylethanolamine C39:1
Sphingomyelin C33:1	Phosphatidylcholine C36:1	Phosphatidylethanolamine C39:3
Sphingomyelin C34:0	Phosphatidylcholine C36:2	Phosphatidylethanolamine C39:6
Sphingomyelin C34:1	Phosphatidylcholine C36:3	Phosphatidylethanolamine C39:7
Sphingomyelin C34:2	Phosphatidylcholine C36:4	Phosphatidylethanolamine C40:6
Sphingomyelin C35:1	Phosphatidylcholine C36:6	Phosphatidylethanolamine C40:7
Sphingomyelin C36:0	Phosphatidylcholine C37:0	
Sphingomyelin C38:0	Phosphatidylcholine C37:4	
Sphingomyelin C38:1	Phosphatidylcholine C37:5	
Sphingomyelin C38:4	Phosphatidylcholine C37:7	
Sphingomyelin C38:5	Phosphatidylcholine C38:0	
Sphingomyelin C39:0	Phosphatidylcholine C38:1	
Sphingomyelin C39:4	Phosphatidylcholine C38:2	
Sphingomyelin C39:5	Phosphatidylcholine C38:3	
Sphingomyelin C40:0	Phosphatidylcholine C38:4	
Sphingomyelin C40:1	Phosphatidylcholine C38:6	

Sphingomyelin C40:2	Phosphatidylcholine C39:0	
Sphingomyelin C41:4	Phosphatidylcholine C39:4	
Sphingomyelin C41:5	Phosphatidylcholine C39:7	
Sphingomyelin C42:0	Phosphatidylcholine C40:0	
Sphingomyelin C42:2	Phosphatidylcholine C40:1	
**Triacylglycerols**	Phosphatidylcholine C40:2	
Triacylglycerol C54:3	Phosphatidylcholine C40:3	
Triacylglycerol C59:0	Phosphatidylcholine C40:4	
Triacylglycerol C61:5	Phosphatidylcholine C40:6	
Triacylglycerol C62:0	Phosphatidylcholine C40:7	
Triacylglycerol C62:1	Phosphatidylcholine C41:0	
	Phosphatidylcholine C41:4	
	Phosphatidylcholine C44:4	
	Phosphatidylcholine C48:4	
