# Supplementary material for: Induced Pluripotent Stem Cells Show Metabolomic Differences to Embryonic Stem Cells in Polyunsaturated Phosphatidylcholines and Primary Metabolism
Source: PLoS One. 2012 Oct 15;7(10):e46770. doi: 10.1371/journal.pone.0046770 (PMC3471894; doi:10.1371/journal.pone.0046770)
Supplement: Table S2 — Identified metabolites in m15 and stem cell cultures using gas chromatography-time of flight mass spectrometry and BinBase annotations, and liquid chromatography-quadrupole time of flight mass spectrometry and Metlin and LipidBlast annotations. Significance levels are given as one-way ANOVA p-values. (PDF) [file pone.0046770.s004.pdf]

Name	PubChem Number	Method	Database	m15 - Average Normalized Intensity (±SD)	iPSC - Average Normalized Intensity (±SD)	mESC - Average Normalized Intensity (±SD)	iPSC/m15		mESC/m15		mESC/iPSC	
							p	Fold Change	p	Fold Change	p	Fold Change
1-monostearin	24699	GCTOF	BinBase	330±285	261±235	318±159	0.6719	0.79	0.9354	0.96	0.6604	1.22
3-phosphoglycerate	724	GCTOF	BinBase	1341±175	1736±1178	3601±2621	0.4800	1.29	0.0906	2.68	0.1497	2.07
5'-Methylthioadenosine	439176	GCTOF	BinBase	599±253	419±110	732±305	0.1469	0.70	0.4758	1.22	0.0429	1.75
5-methoxytryptamine	1833	GCTOF	BinBase	1121±916	673±193	991±242	0.2689	0.60	0.7682	0.88	0.0379	1.47
Adenosine	60961	GCTOF	BinBase	382±216	351±160	547±558	0.7888	0.92	0.5543	1.43	0.4280	1.56
adenosine-5-phosphate	6083	GCTOF	BinBase	16113±7075	6348±460	8496±1791	0.0078	0.39	0.0479	0.53	0.0189	1.34
adipic acid	196	GCTOF	BinBase	373±163	609±227	547±243	0.0845	1.63	0.2205	1.47	0.6715	0.90
Alanine	5950	GCTOF	BinBase	165545±14756	37578±9669	52442±30178	0.0000	0.23	0.0001	0.32	0.2803	1.40
aminomalonic acid	100714	GCTOF	BinBase	2675±2353	592±392	1122±1209	0.0595	0.22	0.2256	0.42	0.3345	1.89
arachidic acid	10467	GCTOF	BinBase	1461±378	1950±1089	2432±1682	0.3671	1.33	0.2436	1.66	0.5798	1.25
Asparagine	236	GCTOF	BinBase	2822±571	809±46	3118±3589	0.0000	0.29	0.8603	1.10	0.1456	3.85
aspartic acid	5960	GCTOF	BinBase	85607±12531	30781±8726	24100±14152	0.0000	0.36	0.0001	0.28	0.3608	0.78
benzoic acid	243	GCTOF	BinBase	5073±2304	5310±3304	5843±4168	0.8955	1.05	0.7270	1.15	0.8180	1.10
beta-alanine	239	GCTOF	BinBase	12104±4290	5912±571	4928±1660	0.0064	0.49	0.0082	0.41	0.2035	0.83
beta-glycerolphosphate	2526	GCTOF	BinBase	236±170	156±40	267±101	0.2888	0.66	0.7306	1.13	0.0341	1.72
capric acid	2969	GCTOF	BinBase	1759±1127	1682±1307	2550±2106	0.9203	0.96	0.4804	1.45	0.4238	1.52
Cellobiotol	160514	GCTOF	BinBase	812±229	654±352	1125±756	0.4128	0.81	0.4011	1.39	0.2041	1.72
Cholesterol	5997	GCTOF	BinBase	218461±155566	157675±36525	216091±39259	0.3736	0.72	0.9745	0.99	0.0310	1.37
citric acid	311	GCTOF	BinBase	13695±5069	11073±647	12708±6331	0.2364	0.81	0.7924	0.93	0.5408	1.15
conduritol-beta-epoxide	119054	GCTOF	BinBase	4336±886	2869±507	3120±2009	0.0072	0.66	0.2509	0.72	0.7725	1.09
Creatinine	588	GCTOF	BinBase	14282±14341	11605±10574	16638±20296	0.7294	0.81	0.8374	1.16	0.6084	1.43
Cysteine	594	GCTOF	BinBase	2645±493	646±352	636±517	0.0000	0.24	0.0002	0.24	0.9727	0.99
cytidine-5'-diphosphate	290	GCTOF	BinBase	363±183	821±761	515±438	0.2250	2.26	0.4935	1.42	0.4492	0.63
dehydroabietic acid	94391	GCTOF	BinBase	1428±877	1465±1190	2231±1900	0.9550	1.03	0.4158	1.56	0.4345	1.52
Dodecane	8182	GCTOF	BinBase	458±191	494±45	571±286	0.6641	1.08	0.4831	1.25	0.5259	1.16
Dodecanol	8193	GCTOF	BinBase	449±185	1441±2356	571±463	0.3767	3.21	0.5997	1.27	0.4414	0.40
elaidic acid	5282749	GCTOF	BinBase	407±52	291±144	522±377	0.1244	0.72	0.5201	1.28	0.1976	1.79
Erythritol	222285	GCTOF	BinBase	1474±222	436±136	631±202	0.0000	0.30	0.0002	0.43	0.0881	1.45
Fructose	107428	GCTOF	BinBase	164±97	158±71	254±191	0.9036	0.96	0.3755	1.55	0.2791	1.61
fructose 1 phosphate	65246	GCTOF	BinBase	217±49	566±161	528±180	0.0012	2.60	0.0059	2.43	0.7257	0.93
fructose-6-phosphate	69507	GCTOF	BinBase	915±471	494±260	721±528	0.0920	0.54	0.5563	0.79	0.3753	1.46
fumaric acid	444972	GCTOF	BinBase	2391±285	2080±255	2181±470	0.0881	0.87	0.4161	0.91	0.6615	1.05
GABA	119	GCTOF	BinBase	27455±8697	14469±7559	16300±5398	0.0264	0.53	0.0408	0.59	0.6619	1.13
galactose-6-phosphate	99058	GCTOF	BinBase	353±247	231±98	168±23	0.2943	0.66	0.1349	0.48	0.1965	0.73
Glucose	5793	GCTOF	BinBase	1043±314	2336±1563	2201±1327	0.1048	2.24	0.0942	2.11	0.8821	0.94
glucose-1-phosphate	65533	GCTOF	BinBase	1985±1950	2645±1838	1224±1278	0.5784	1.33	0.4862	0.62	0.1799	0.46
glucose-6-phosphate	5958	GCTOF	BinBase	587±380	241±112	319±243	0.0608	0.41	0.2210	0.54	0.4980	1.32
glutamic acid	33032	GCTOF	BinBase	87873±78030	36122±29399	54034±54938	0.1643	0.41	0.4507	0.61	0.5057	1.50
Glutamine	5961	GCTOF	BinBase	14153±12335	1233±354	2767±1496	0.0291	0.09	0.0746	0.20	0.0365	2.24
Glutathione	124886	GCTOF	BinBase	1129±869	258±121	362±265	0.0367	0.23	0.0960	0.32	0.4087	1.40
glyceric acid	439194	GCTOF	BinBase	611±224	601±339	850±647	0.9570	0.98	0.4575	1.39	0.4324	1.41
glycerol-alpha-phosphate	754	GCTOF	BinBase	15820±8266	8536±3528	16354±7966	0.0803	0.54	0.9197	1.03	0.0573	1.92

Glycine	750	GCTOF	BinBase	649509±49332	105702±26666	132745±91896	0.0000	0.16	0.0000	0.20	0.5055	1.26
glycolic acid	757	GCTOF	BinBase	1056±163	1191±484	1644±788	0.5663	1.13	0.1407	1.56	0.2709	1.38
Guanosine	6802	GCTOF	BinBase	128±26	453±372	356±290	0.0853	3.55	0.1172	2.79	0.6482	0.79
hexuronic acid	19770757	GCTOF	BinBase	963±135	857±46	619±372	0.1038	0.89	0.0880	0.64	0.1502	0.72
Hydroxylamine	787	GCTOF	BinBase	6668±917	7352±1323	8498±4782	0.3559	1.10	0.4251	1.27	0.5844	1.16
idonic acid	193325	GCTOF	BinBase	386±121	261±60	290±61	0.0515	0.68	0.1527	0.75	0.4374	1.11
inosine 5'-monophosphate	8582	GCTOF	BinBase	423±239	149±40	198±118	0.0207	0.35	0.0950	0.47	0.3593	1.33
Inositol	892	GCTOF	BinBase	151639±57783	113939±31668	115822±95962	0.2015	0.75	0.4950	0.76	0.9646	1.02
inositol-4-monophosphate	440043	GCTOF	BinBase	409±162	422±43	545±181	0.8558	1.03	0.2454	1.33	0.1369	1.29
Inulobiose	439552	GCTOF	BinBase	6993±8844	5413±5225	8684±10418	0.7206	0.77	0.7889	1.24	0.5146	1.60
Isoleucine	6306	GCTOF	BinBase	25573±4980	7856±1248	12447±2847	0.0000	0.31	0.0009	0.49	0.0059	1.58
lactic acid	612	GCTOF	BinBase	10729±9797	11157±6900	11443±10471	0.9341	1.04	0.9141	1.07	0.9577	1.03
lauric acid	3893	GCTOF	BinBase	19465±16335	16963±16378	30809±28148	0.8063	0.87	0.4582	1.58	0.3338	1.82
Leucine	6106	GCTOF	BinBase	14434±15000	9218±1162	7130±6331	0.4130	0.64	0.3452	0.49	0.4442	0.77
Levoglucosan	2724705	GCTOF	BinBase	492±117	383±98	396±107	0.1251	0.78	0.2098	0.80	0.8390	1.03
Lysine	5962	GCTOF	BinBase	4925±2687	1735±716	2504±929	0.0201	0.35	0.0933	0.51	0.1547	1.44
malic acid	222656	GCTOF	BinBase	4534±2518	5496±721	4549±1179	0.3907	1.21	0.9905	1.00	0.1348	0.83
Mannitol	6251	GCTOF	BinBase	875±665	3008±2401	4342±2333	0.0883	3.44	0.0127	4.96	0.3771	1.44
methanolphosphate	13130	GCTOF	BinBase	1267±1193	1173±801	1050±494	0.8799	0.93	0.7164	0.83	0.7711	0.89
Methionine	6137	GCTOF	BinBase	3596±508	1029±292	1227±316	0.0000	0.29	0.0000	0.34	0.3084	1.19
methylhexadecanoic acid	10465	GCTOF	BinBase	1733±494	1687±586	2352±1133	0.8927	0.97	0.2955	1.36	0.2401	1.39
monopalmitin-1-glyceride	14900	GCTOF	BinBase	280±61	232±61	278±137	0.2304	0.83	0.9837	0.99	0.4724	1.20
myristic acid	11005	GCTOF	BinBase	959±858	1710±837	1117±1401	0.1770	1.78	0.8345	1.17	0.4063	0.65
N-acetyl-D-mannosamine	65150	GCTOF	BinBase	212±41	206±74	272±154	0.8842	0.97	0.4181	1.29	0.3708	1.32
Octadecanol	8221	GCTOF	BinBase	864±606	665±168	795±440	0.4557	0.77	0.8411	0.92	0.5176	1.20
oleic acid	445639	GCTOF	BinBase	1166±214	880±539	1394±1017	0.2969	0.75	0.6381	1.19	0.3102	1.58
Ornithine	6262	GCTOF	BinBase	6873±5318	1626±158	1989±450	0.0372	0.24	0.0749	0.29	0.0959	1.22
Oxoproline	7405	GCTOF	BinBase	827014±550035	160555±78198	219556±80361	0.0158	0.19	0.0403	0.27	0.2496	1.37
palmitic acid	985	GCTOF	BinBase	8543±561	8676±2346	12047±6610	0.9050	1.02	0.2715	1.41	0.2705	1.39
palmitoleic acid	445638	GCTOF	BinBase	1722±1097	1357±701	1403±387	0.5197	0.79	0.5570	0.81	0.8994	1.03
pantothenic acid	6613	GCTOF	BinBase	2048±679	787±150	899±440	0.0016	0.38	0.0131	0.44	0.5705	1.14
pelargonic acid	8158	GCTOF	BinBase	19401±18271	15875±15647	20025±14962	0.7378	0.82	0.9544	1.03	0.6658	1.26
Phenylalanine	6140	GCTOF	BinBase	11395±3813	3359±466	4582±1135	0.0006	0.29	0.0050	0.40	0.0381	1.36
Phosphate	1004	GCTOF	BinBase	177637±153036	200431±93979	319440±205201	0.7679	1.13	0.2506	1.80	0.2330	1.59
phosphoethanolamine	1015	GCTOF	BinBase	7216±4396	15326±4445	19577±7288	0.0143	2.12	0.0117	2.71	0.2631	1.28
phosphoric acid	1004	GCTOF	BinBase	151376±151576	278622±64205	324620±308426	0.0929	1.84	0.2923	2.14	0.7273	1.17
phthalic acid	1017	GCTOF	BinBase	2896±257	3225±1554	3900±2375	0.6535	1.11	0.3746	1.35	0.5835	1.21
p-hydroquinone	785	GCTOF	BinBase	595±299	588±415	966±842	0.9746	0.99	0.3810	1.62	0.3558	1.64
Proline	145742	GCTOF	BinBase	70985±10802	11055±1366	13025±4927	0.0000	0.16	0.0000	0.18	0.3687	1.18
propane-1,3-diol	10442	GCTOF	BinBase	16609±21707	15552±16420	9382±11328	0.9286	0.94	0.5278	0.56	0.4965	0.60
Putrescine	1045	GCTOF	BinBase	23229±15022	7341±937	10687±2679	0.0281	0.32	0.1034	0.46	0.0181	1.46
pyrazine 2,5-dihydroxy	15532987	GCTOF	BinBase	2969±3518	1895±1840	2760±3334	0.5302	0.64	0.9256	0.93	0.5976	1.46
pyrophosphate 1	1023	GCTOF	BinBase	313041±78693	105550±84258	170882±171869	0.0024	0.34	0.1311	0.55	0.4303	1.62

pyruvic acid	1060	GCTOF	BinBase	2038±494	1330±494	1891±789	0.0420	0.65	0.7317	0.93	0.1833	1.42
Ribitol	827	GCTOF	BinBase	1296±393	663±169	1007±601	0.0058	0.51	0.3944	0.78	0.2094	1.52
Ribose	5779	GCTOF	BinBase	257±58	1631±1629	642±503	0.0947	6.34	0.1276	2.50	0.2273	0.39
ribose-5-phosphate	77982	GCTOF	BinBase	836±621	641±252	575±273	0.4969	0.77	0.4154	0.69	0.6884	0.90
ribulose-5-phosphate	439184	GCTOF	BinBase	405±138	339±157	433±324	0.4797	0.84	0.8637	1.07	0.5421	1.28
Serine	5951	GCTOF	BinBase	32411±3346	9666±1763	19694±5431	0.0000	0.30	0.0021	0.61	0.0020	2.04
Sorbitol	5780	GCTOF	BinBase	3997±3061	17624±20203	10515±7478	0.1728	4.41	0.1089	2.63	0.4781	0.60
Spermidine	1102	GCTOF	BinBase	5208±1578	7118±3326	10942±7173	0.2714	1.37	0.1190	2.10	0.2711	1.54
stearic acid	5281	GCTOF	BinBase	88086±7341	91400±32271	126872±72093	0.8285	1.04	0.2656	1.44	0.3040	1.39
succinic acid	1110	GCTOF	BinBase	1689±701	995±179	1044±98	0.0427	0.59	0.0758	0.62	0.6023	1.05
Sucrose	5988	GCTOF	BinBase	138±62	531±952	150±63	0.3860	3.83	0.7793	1.08	0.3995	0.28
Taurine	1123	GCTOF	BinBase	2916±1211	924±721	1818±2196	0.0080	0.32	0.3563	0.62	0.3689	1.97
Threitol	169019	GCTOF	BinBase	233±42	145±21	157±44	0.0014	0.62	0.0231	0.67	0.5611	1.08
threonic acid	439535	GCTOF	BinBase	241±91	150±45	210±97	0.0586	0.62	0.6156	0.87	0.2119	1.40
Threonine	6288	GCTOF	BinBase	64497±7352	9297±1414	18413±7383	0.0000	0.14	0.0000	0.29	0.0152	1.98
trans-4-hydroxyproline	5810	GCTOF	BinBase	1295±292	426±67	725±255	0.0001	0.33	0.0111	0.56	0.0208	1.70
Triethanolamine	7618	GCTOF	BinBase	402±252	460±419	759±608	0.7921	1.15	0.2598	1.89	0.3603	1.65
Tryptophan	6305	GCTOF	BinBase	5299±2798	1985±283	2547±584	0.0171	0.37	0.0635	0.48	0.0654	1.28
Tyrosine	6057	GCTOF	BinBase	23171±7321	6994±525	10528±2311	0.0004	0.30	0.0062	0.45	0.0051	1.51
UDP GlcNAc	445675	GCTOF	BinBase	2768±342	1161±81	1978±438	0.0000	0.42	0.0130	0.71	0.0014	1.70
UDP-glucuronic acid	17473	GCTOF	BinBase	4558±2786	2252±1229	3159±2503	0.0991	0.49	0.4276	0.69	0.4520	1.40
Uracil	1174	GCTOF	BinBase	1235±288	8301±7272	6406±5068	0.0599	6.72	0.0523	5.19	0.6358	0.77
Urea	1176	GCTOF	BinBase	1413±1507	1254±1742	2174±2266	0.8759	0.89	0.5495	1.54	0.4650	1.73
Valine	6287	GCTOF	BinBase	31162±13918	9732±1053	14623±3288	0.0042	0.31	0.0323	0.47	0.0071	1.50
Xanthine	1188	GCTOF	BinBase	141±18	233±62	265±156	0.0110	1.65	0.1135	1.89	0.6491	1.14
Xylitol	6912	GCTOF	BinBase	1359±478	1486±797	1329±742	0.7640	1.09	0.9412	0.98	0.7460	0.89
lysoPC 14:0	460604	HILIC Pos	LipidBlastPos	5353±1205	2579±1179	4942±1963	0.0024	0.48	0.6714	0.92	0.0299	1.92
lysoPC 16:0	460602	HILIC Pos	LipidBlastPos	169080±31167	77996±17303	152239±11477	0.0001	0.46	0.2426	0.90	0.0000	1.95
lysoPC 16:1	24779461	HILIC Pos	LipidBlastPos	14569±1853	7186±5291	11259±7217	0.0091	0.49	0.3021	0.77	0.2909	1.57
lysoPC 18:0	497299	HILIC Pos	LipidBlastPos	153797±29090	103673±8418	196096±34611	0.0023	0.67	0.0449	1.28	0.0001	1.89
lysoPC 18:1	16081932	HILIC Pos	LipidBlastPos	103353±27458	38078±24885	60520±30134	0.0015	0.37	0.0277	0.59	0.1898	1.59
lysoPE 18:1	9547071	HILIC Pos	LipidBlastPos	16766±6949	3942±2167	3540±961	0.0015	0.24	0.0010	0.21	0.6870	0.90
PC 30:0	24778679	HILIC Pos	LipidBlastPos	834905±207960	585790±160464	905219±182321	0.0425	0.70	0.5474	1.08	0.0092	1.55
PC 32:0	452110	HILIC Pos	LipidBlastPos	729113±235729	1018269±364907	1238916±244640	0.1341	1.40	0.0043	1.70	0.2468	1.22
PC 32:1	52922440	HILIC Pos	LipidBlastPos	1097793±405733	925087±159929	1024956±337771	0.3549	0.84	0.7424	0.93	0.5275	1.11
PC 32:2	24778764	HILIC Pos	LipidBlastPos	104357±46329	79311±21189	89445±33837	0.2562	0.76	0.5386	0.86	0.5480	1.13
PC 34:1	24778933	HILIC Pos	LipidBlastPos	2107851±266838	1861708±281072	1757321±381403	0.1508	0.88	0.0949	0.83	0.6012	0.94
PC 34:2	24778935	HILIC Pos	LipidBlastPos	903864±133722	680782±132597	652131±185970	0.0158	0.75	0.0226	0.72	0.7649	0.96
PC 36:2	10350317	HILIC Pos	LipidBlastPos	1711074±224685	1327366±171296	1161596±204974	0.0077	0.78	0.0013	0.68	0.1595	0.88
PC 36:3	52922727	HILIC Pos	LipidBlastPos	312557±63314	376365±53278	477463±114202	0.0882	1.20	0.0114	1.53	0.0778	1.27
PC 36:4	24779073	HILIC Pos	LipidBlastPos	421529±66275	704331±62613	991412±110446	0.0000	1.67	0.0000	2.35	0.0002	1.41
PC 38:2	52923113	HILIC Pos	LipidBlastPos	113786±14611	114670±38607	85323±28810	0.9592	1.01	0.0563	0.75	0.1665	0.74
PC 38:3	52923231	HILIC Pos	LipidBlastPos	93578±32176	194655±34841	228616±61801	0.0004	2.08	0.0008	2.44	0.2682	1.17
PC 38:4	52923291	HILIC Pos	LipidBlastPos	226511±46431	718700±31112	927458±76090	0.0000	3.17	0.0000	4.09	0.0001	1.29
PC 38:5	52923293	HILIC Pos	LipidBlastPos	346695±102732	504700±37155	700345±46931	0.0053	1.46	0.0000	2.02	0.0000	1.39

PC 38:6	24779131	HILIC Pos	LipidBlastPos	128578±69819	188054±27705	260412±43051	0.0811	1.46	0.0028	2.03	0.0061	1.38
PC 40:7	52923693	HILIC Pos	LipidBlastPos	109956±34783	186453±18969	210213±9659	0.0008	1.70	0.0000	1.91	0.0210	1.13
PE 32:1	52924223	HILIC Pos	LipidBlastPos	33499±9812	23845±7145	19251±4833	0.0800	0.71	0.0096	0.57	0.2212	0.81
PE 34:1	9546802	HILIC Pos	LipidBlastPos	154870±35801	147803±28337	93463±22277	0.7125	0.95	0.0051	0.60	0.0042	0.63
PE 34:2	52924899	HILIC Pos	LipidBlastPos	96388±16357	60895±22764	42069±12180	0.0112	0.63	0.0001	0.44	0.1044	0.69
PE 36:2	9546757	HILIC Pos	LipidBlastPos	367443±90106	287917±63334	152970±37349	0.1074	0.78	0.0003	0.42	0.0012	0.53
PE 36:3	52924364	HILIC Pos	LipidBlastPos	61078±13278	42427±11671	40279±12193	0.0349	0.69	0.0241	0.66	0.7616	0.95
PE 36:4	52924875	HILIC Pos	LipidBlastPos	84357±34846	82756±15387	104652±26984	0.9200	0.98	0.2857	1.24	0.1149	1.26
PE 38:4	52924644	HILIC Pos	LipidBlastPos	217461±62464	303210±19925	364570±49287	0.0094	1.39	0.0011	1.68	0.0179	1.20
PE 38:5	52924645	HILIC Pos	LipidBlastPos	168743±35887	150943±23408	167691±34871	0.3328	0.89	0.9599	0.99	0.3517	1.11
PE 38:6	52924862	HILIC Pos	LipidBlastPos	53276±21503	69057±14108	79311±21551	0.1637	1.30	0.0626	1.49	0.3525	1.15
PE 40:7	52924844	HILIC Pos	LipidBlastPos	74673±17831	82156±2066	77373±4850	0.3312	1.10	0.7279	1.04	0.0505	0.94
plasmenyl-PC 34:1	24779384	HILIC Pos	LipidBlastPos	56320±1398	35464±2466	37972±6400	0.0011	0.63	0.0138	0.67	0.6566	1.07
plasmenyl-PC 36:4	24779388	HILIC Pos	LipidBlastPos	22757±11370	130015±16964	199522±31453	0.0000	5.71	0.0000	8.77	0.0008	1.53
plasmenyl-PE 34:1	52925128	HILIC Pos	LipidBlastPos	128478±45980	65709±11252	52534±10137	0.0087	0.51	0.0027	0.41	0.0589	0.80
plasmenyl-PE 34:2	52925127	HILIC Pos	LipidBlastPos	52326±9051	20318±5929	20477±5153	0.0000	0.39	0.0000	0.39	0.9614	1.01
plasmenyl-PE 36:2	52925059	HILIC Pos	LipidBlastPos	121275±48879	51985±12384	39172±7206	0.0072	0.43	0.0022	0.32	0.0533	0.75
plasmenyl-PE 36:4	52925126	HILIC Pos	LipidBlastPos	216509±72775	353851±32283	408149±45979	0.0018	1.63	0.0003	1.89	0.0395	1.15
plasmenyl-PE 38:4	9547058	HILIC Pos	LipidBlastPos	136871±29801	179484±27360	198979±23960	0.0274	1.31	0.0026	1.45	0.2185	1.11
plasmenyl-PE 38:6	5283497	HILIC Pos	LipidBlastPos	116339±51341	147347±38833	173049±46728	0.2653	1.27	0.0733	1.49	0.3245	1.17
plasmenyl-PE 40:6	42607458	HILIC Pos	LipidBlastPos	96628±29545	100047±27106	118224±32115	0.8388	1.04	0.2533	1.22	0.3143	1.18
SM 32:1	11433862	HILIC Pos	LipidBlastPos	30414±5287	25244±3821	36860±6299	0.0809	0.83	0.0838	1.21	0.0031	1.46
SM 34:1	9939941	HILIC Pos	LipidBlastPos	766916±538287	807358±370629	964904±407867	0.8825	1.05	0.4891	1.26	0.4997	1.20
SM 34:2	52931143	HILIC Pos	LipidBlastPos	95114±14177	74480±10630	116910±23727	0.0172	0.78	0.0822	1.23	0.0025	1.57
SM 36:2	6443882	HILIC Pos	LipidBlastPos	17477±4301	14947±1358	16803±3104	0.1993	0.86	0.7619	0.96	0.2092	1.12
Acetylcarnitine	7045767	HILIC Pos	Metlin	41161±27733	19204±7620	32543±29444	0.0910	0.47	0.6132	0.79	0.3079	1.69
Betaine	247	HILIC Pos	Metlin	69668±49909	101659±53369	57760±53309	0.3087	1.46	0.6980	0.83	0.1845	0.57
Butyryl carnitine	213144	HILIC Pos	Metlin	6675±1180	2576±702	5295±3400	0.0000	0.39	0.3698	0.79	0.0840	2.06
Carnitine	10917	HILIC Pos	Metlin	49880±39128	32988±19821	50112±46303	0.3677	0.66	0.9927	1.00	0.4244	1.52
Creatine	586	HILIC Pos	Metlin	165161±149386	256790±236298	442640±462043	0.4407	1.55	0.1919	2.68	0.4010	1.72
Pantothenic Acid	6613	HILIC Pos	Metlin	16266±2975	9255±1452	11782±7345	0.0004	0.57	0.1958	0.72	0.4277	1.27
Sphingosine	5280335	HILIC Pos	Metlin	37105±15123	28978±3820	40518±6732	0.2307	0.78	0.6244	1.09	0.0044	1.40
Taurine	1123	HILIC Pos	Metlin	324287±177289	278594±180009	356678±325883	0.6672	0.86	0.8349	1.10	0.6186	1.28
Adenosine	60961	HILIC Pos	NIST	3048±725	6457±5052	7208±1914	0.1333	2.12	0.0011	2.36	0.7886	1.12
5'-Methylthioadenosine	439176	RP Pos	Metlin	27307±1598	18030±992	33961±4076	0.0000	0.66	0.0094	1.24	0.0000	1.88
Pantothenic Acid	6613	RP Pos	Metlin	17423±1598	9608±992	13820±5780	0.0000	0.55	0.2128	0.79	0.1090	1.44
