# Supplementary material for: Induced Pluripotent Stem Cells Show Metabolomic Differences to Embryonic Stem Cells in Polyunsaturated Phosphatidylcholines and Primary Metabolism
Source: PLoS One. 2012 Oct 15;7(10):e46770. doi: 10.1371/journal.pone.0046770 (PMC3471894; doi:10.1371/journal.pone.0046770)
Supplement: Table S3 — Extended information on Materials and Methods : Cell Culture and Preparation; NanoESI-MS Extraction and Analysis; GC-TOF MS Extraction and Analysis; LC-QTOF MS Extraction and Analysis; MetaMapp Network Visualization. (PDF) [file pone.0046770.s005.pdf]

Extended information on Materials and Methods

Cell Culture and Preparation

The m15 parental fibroblast cells were isolated from E14.5 CF-1 embryos and maintained in MEF medium (10% FBS (HyClone), 100 U/ml penicillin, 100 U/ml streptomycin, 1xGlutamax, 1xNEAA (Gibco) in high glucose DMEM). iPSCs were derived through two rounds of transduction of m15 parental fibroblast cells with a mixture of pMXs based retroviruses encoding the four genes Oct4, Sox2, Klf4, and c-Myc. Detailed MEF isolation and transduction protocols are previously described (1). MEF medium was changed daily for the first seven days, and seven days post-transduction the cells were placed on an irradiated MEF feeder layer. Media was subsequently changed daily with mESC medium (15%FBS (HyClone), 100 U/ml penicillin, 100 U/ml streptomycin, 1mM sodium pyruvate, 0.1 mM b-mercaptoethanol, 1xGlutamax, 1xNEAA (Gibco),with 1000 U/mlLIF (ESGRO,Chemicon) in high glucose DMEM) until twenty one days post-transduction, when colonies were clearly visible. Colonies with typical mESC morphology were manually picked and enzymatically passaged. Control mESCs and m15 fibroblast cells were enzymatically passaged and maintained in mESC medium. To prepare cell pellets for metabolite extraction, all cells were harvested by trypsinization, washed three times with cold PBS, divided into one million cell aliquots, and flash frozen in liquid N_2. Cell pellets were stored at -80^0C prior to extraction.

NanoESI-MS Extraction and Analysis

Three replicates from each cell type were used for nanoESI-MS analysis. Frozen cell pellets were thawed on ice, and 225 μL of methanol was added to each sample. Samples were vortexed for 10 sec, received 750 μL of t-butyl methyl ether, and were placed in an orbital shaker for 5 minutes at 4^0C. Samples received an additional 187.5 μL of H_2O, were vortexed for 20 sec, and were centrifuged for 2 min at 14,000 rcf. Supernatant was divided into two 360 μL aliquots and lyophilized to dryness. Lyophilized material was resuspended in 100 μL of 9:1 methanol/chloroform, and 10 μL of resuspended material was diluted with 90 μL of 9:1 methanol/chloroform containing 7.5 mM ammonium acetate. Sample material was placed into an Eppendorf twin.tec 96-well PCR plate sealed with an Eppendorf PCR adhesive foil plate cover.

Mass spectrometry analysis was performed with a LTQ linear ion trap mass spectrometer (ThermoFisher Scientific, San Jose, CA) coupled to an Advion NanoMate chip based nanoelectrospray ionization source (Advion Biosciences Inc., Ithaca, NY). Sample material was analyzed in positive ion mode with an ionization voltage of 2.0 kV and 0.4 psi gas pressure. MS data spanning 350-1100 m/z was acquired for 1 min for each replicate. MS/MS acquisition was applied to pooled samples representative of each sample condition and entailed a 6 sec MS scan to determine appropriate precursor ions followed by a 10 min data dependent acquisition. MS/MS experiments were performed for 350-450, 450-750, 750-850, and 850-1100 m/z ranges to increase metabolite coverage.

Raw data files were imported into GeneData Expressionist Refiner MS v6.2.0 software (GeneData, Basel, Switzerland) and processed yielding an aligned peak list with ion intensities. The peak list was imported into Statistica 9.0 software (StatSoft, Tulsa, OK) for PLS analysis. For MS/MS library annotation, raw data files were converted to mascot generic format (mgf) using Thermo ExtractMSn software and the DeconMSn programs (2). The NIST MS Search program (National Institutes of Standards and Technology, Gaithersburg, MD) was used to compare MS/MS data in mgf format to LipidBlast, an in-house library of lipid structure MS/MS spectra. LipidBlast extends the structural diversity included in LipidMAPS with additional lipid classes and by including hypothetical odd-chain fatty acyl groups for chemical scaffolds of 29 lipid classes. It models 200,000 lipid MS/MS spectra which were used to annotate individual acyl chain lengths and extent of saturation as well as determine the overall lipid class. All annotations were manually confirmed.

GC-TOF MS Extraction and Analysis

Six replicates, obtained from two independent cell cultures from each cell type conducted three months apart were used for GC-TOF analysis. Cell pellets were extracted, derivatized, and analyzed as reported previously (3,4). Samples were resuspended in 1 mL of 3:3:2 acetonitrile:isopropanol:H_2O and sonicated for 5 minutes with a VWR Model 50HT ultrasonic bath (VWR International Inc., Bridgeport, NJ). Suspended material was divided into two aliquots, centrifuged for 5 min at 14,000 rcf, and lyophilized to dryness. Lyophilized material was resuspended in 500 µL of 1:1 acetonitrile:H_2O and centrifuged for 2 min at 14,000 rcf to remove membrane lipids and triglycerides. Supernatant was collected and lyophilized. A set of thirteen C8-C30 fatty acid methyl ester internal standards were added and samples were derivatized by the addition of 10 µL methoxyamine hydrochloride in pyridine followed by 90 µL MSTFA for trimethylsilylation of acidic protons.

A Gerstel MPS2 automatic liner exchange system (Gerstel GMBH & Co.KG, Mülheim an der Ruhr, Germany) was used to inject 0.5 µL of sample at 50ºC (ramped to 250ºC) in splitless mode with 25 sec splitless time. Analytes were separated using an Agilent 6890 gas chromatograph (Agilent Technologies, Santa Clara, CA) equipped with a 30 m long, 0.25 mm i.d. Rtx5Sil-MS column with 0.25 mm 5% diphenyl film and an additional 10 m integrated guard column (Restek, Bellefonte PA). Chromatography was performed with a constant flow of 1 mL/min while ramping the oven temperature from 50ºC to 330ºC with 22 min total run time. MS was performed with a Leco Pegasus IV time of flight mass spectrometer (Leco Corporation, St. Joseph, MI) with a 280ºC transfer line temperature, an electron ionization voltage of 270eV, and an ion source temperature of 250ºC. Mass spectra were acquired between 85–500 m/z with a scan rate of 17 spectra/sec.

Result files were exported to our servers and further processed by our metabolomics BinBase database (5). All database entries in BinBase were matched against the Fiehn mass spectral library of 1200 authentic metabolite spectra using retention index and mass spectrum information or the NIST05 commercial library. Peak heights of quantifier ions defined for each metabolite in BinBase were normalized to the average sum of intensities of all replicates in an experimental condition divided by the sample sum of intensities. External 5-point calibration curves established with quality control mixtures containing 30 metabolites controlled for instrument sensitivity.

LC-QTOF MS Extraction and Analysis

Six replicates, obtained from two independent cell cultures from each cell type conducted three months apart were used for LC-QTOF MS analysis. Frozen cell pellets were thawed on ice, and 1000 µL of 3:1 methanol:H_2O extraction solvent was added to each sample. Samples were vortexed for 20 sec, sonicated for 5 min, and centrifuged for 5 min at 14,000 rcf. Supernatant was divided into two 500 µL aliquots and lyophilized to dryness. Lyophilized material was redissolved in 100 µL initial LC gradient solvent and analyzed within 24 hours.

An Agilent 1200 Series HPLC system, equipped with an autosampler and a thermostatted column compartment maintained at 4^0C and 40^0C respectively, was used for chromatographic separation of 5 µL sample material. HILIC analysis was performed with a Waters 1.7 µm Acquity BEH HILIC 2.1x150 mm column (Waters Corporation, Milford, MA). Mobile phase consisted of H_2O with 5 mM ammonium acetate and 0.2% acetic acid (A) and 9:1 acetonitrile:H_2O with 5 mM ammonium acetate and 0.2% acetic acid (B). The gradient method was: 0-4 min – 100% B, 4-12 min – linear gradient to 45% B, and 12-20 min – 45% B. The column was re-equilibrated for 20 min following each sample run, and flow rate was constant at 0.25 mL/min throughout the gradient method and re-equilibration. RP analysis was performed with an Agilent 1.8 µm Zorbax Eclipse Plus C18 2.1x150 mm column. Mobile phase consisted of H_2O with 0.1% formic acid (A) and acetonitrile with 0.1% formic acid (B). The gradient method was: 0-4 min – 5% B, 4-12 min – linear gradient to 100% B, and 12-20 min – 100% B. Column flow rate and re-equilibration duration were identical to the HILIC method.

LC eluents were analyzed with an Agilent 6530 accurate-mass Q-TOF mass spectrometer equipped with an Agilent Jet Stream ESI source in positive ion mode. MS and MS/MS data was collected with a 0.25 sec scan rate in both profile and centroid modes, and mass calibration was maintained by

constant infusion of reference ions at 121.0509 and 922.0098 m/z. MS/MS data was generated utilizing data dependent MS/MS triggering with dynamic exclusion. Parent ions, with a minimum 1000 signal intensity, were isolated with a 4 m/z isolation width, and a variable collision energy was applied based on parent ion m/z (10 eV + 0.02 eV * ion m/z). Ions were excluded from data dependent MS/MS analysis for 30 sec following acquisition of 3 spectra.

Raw data files were converted to mzXML format with the Trapper mzXML conversion software and processed with the MZmine v2.0 data processing software to generate an aligned peak list with ion intensities (6). For MS/MS library annotation, raw data files were imported into Agilent Mass Hunter Qual software, and ions with associated MS/MS spectra were extracted with the Find by Auto MS/MS tool. Extracted ions were annotated with the Search Accurate Mass Library tool equipped with the METLIN library. Raw data files were also converted to mgf format with Agilent Mass Hunter Qual software and imported into the NIST MS Search program equipped with the NIST MS/MS library and LipidBlast for MS/MS based annotation. All MS/MS library matches were manually confirmed and any annotation with greater than 1.5 mDa mass error was removed. Annotated structures were matched to the MS level aligned peak list based on mass and retention time to create a matrix of annotated compounds and peak intensities. Annotated ion intensities were normalized to the average sum of intensities of all replicates in an experimental condition divided by the sample sum of intensities. External quality control mixtures containing multiple acylcarnitine structures controlled for chromatography quality and instrument sensitivity.

MetaMapp Network Visualization

Data sets from the GC-TOF MS and LC-QTOF MS platforms were integrated into a single data set. In the case of duplicate annotations, the method with the greatest signal to noise ratio was selected for further data processing. Statistical analysis was performed with Statistica 9.0 software. Molefile encoded chemical structures of all the annotated metabolites were retrieved from the PubChem compound database using compound identifiers (CIDs) and the NCBI Batch Entrez utility (7). The retrieved structures were clustered with a web-based PubChem structural clustering tool producing a pair-wise chemical similarity matrix. The matrix and CID-KEGG ID pairs for metabolites were used as an input in MetaMapp software (available at: http://metamapp.fiehnlab.ucdavis.edu) for generation of Cytoscape network files in simple interaction format (8). A threshold of 0.7 Tanimoto score was used to define the similarity cut-off among metabolites, and an additional similarity threshold of 0.85 Tanimoto score was used for lipid species. A KEGG RPAIR reaction network graph was created using a single-metabolic step neighbor finding algorithm in MetaMapp (9). The final network graphs were imported into Cytoscape and merged into a single network graph. Results of differential statistics generated using Statistica 9.0 software were converted into Cytoscape node attribute files and were imported into Cytoscape. The graph was visualized using a yED organic layout algorithm in Cytoscape. Fold change was mapped to node size, and direction was mapped to node color. Metabolites that did not pass the p-value threshold of <0.05 were not labeled.

REFERENCES

1. Takahashi K, Yamanaka S (2006) Induction of pluripotent stem cells from mouse embryonic and adult fibroblast cultures by defined factors. *Cell* 126:663-676.
2. Mayampurath AM et al. (2008) DeconMSn: a software tool for accurate parent ion monoisotopic mass determination for tandem mass spectra. *Bioinformatics* 24:1021-1023.
3. Fiehn O et al. (2010) Plasma metabolomic profiles reflective of glucose homeostasis in non-diabetic and type 2 diabetic obese African-American women. *PLoS One* 5:e15234.
4. Kind T et al. (2009) FiehnLib: Mass Spectral and Retention Index Libraries for Metabolomics Based on Quadrupole and Time-of-Flight Gas Chromatography/Mass Spectrometry. *Anal Chem* 81:10038-10048.

5. Fiehn O, Wohlgemuth G, Scholz M (2005) Setup and Annotation of Metabolomic Experiments by Integrating Biological and Mass Spectrometric Metadata. *Lect Notes Comput Sci* 3615:224–239.
6. Pluskal T, Castillo S, Villar-Briones A, Orešič M (2010) MZmine 2: Modular framework for processing, visualizing, and analyzing mass spectrometry-based molecular profile data. *BMC Bioinformatics* 11:395.
7. Wang Y et al. (2009) PubChem: a public information system for analyzing bioactivities of small molecules. *Nucleic Acids Res* 37:W623-633.
8. Shannon P et al. (2003) Cytoscape: a software environment for integrated models of biomolecular interaction networks. *Genome Res* 13:2498-2504.
9. Kanehisa M, Goto S, Furumichi M, Tanabe M, Hirakawa M (2010) KEGG for representation and analysis of molecular networks involving diseases and drugs. *Nucleic Acids Res* 38:D355-360.
